# Interleukin-19 Abrogates Experimental Autoimmune Encephalomyelitis by Attenuating Antigen-Presenting Cell Activation

**DOI:** 10.3389/fimmu.2021.615898

**Published:** 2021-03-11

**Authors:** Hiroshi Horiuchi, Bijay Parajuli, Hiroyasu Komiya, Yuki Ogawa, Shijie Jin, Keita Takahashi, Yasu-Taka Azuma, Fumiaki Tanaka, Akio Suzumura, Hideyuki Takeuchi

**Affiliations:** ^1^Department of Neuroimmunology, Research Institute of Environmental Medicine, Nagoya University, Nagoya, Japan; ^2^Department of Neurology and Stroke Medicine, Yokohama City University Graduate School of Medicine, Yokohama, Japan; ^3^Laboratory of Veterinary Pharmacology, Division of Veterinary Science, Osaka Prefecture University Graduate School of Life and Environmental Science, Izumisano, Japan

**Keywords:** interleukin-19, macrophage, antigen presentation, experimental autoimmune encephalomyelitis, multiple sclerosis

## Abstract

Interleukin-19 (IL-19) acts as a negative-feedback regulator to limit proinflammatory response of macrophages and microglia in autocrine/paracrine manners in various inflammatory diseases. Multiple sclerosis (MS) is a major neuroinflammatory disease in the central nervous system (CNS), but it remains uncertain how IL-19 contributes to MS pathogenesis. Here, we demonstrate that IL-19 deficiency aggravates experimental autoimmune encephalomyelitis (EAE), a mouse model of MS, by promoting IL-17-producing helper T cell (Th17 cell) infiltration into the CNS. In addition, IL-19-deficient splenic macrophages expressed elevated levels of major histocompatibility complex (MHC) class II, co-stimulatory molecules, and Th17 cell differentiation-associated cytokines such as IL-1β, IL-6, IL-23, TGF-β1, and TNF-α. These observations indicated that IL-19 plays a critical role in suppression of MS pathogenesis by inhibiting macrophage antigen presentation, Th17 cell expansion, and subsequent inflammatory responses. Furthermore, treatment with IL-19 significantly abrogated EAE. Our data suggest that IL-19 could provide significant therapeutic benefits in patients with MS.

## Introduction

Multiple sclerosis (MS) and experimental autoimmune encephalomyelitis (EAE), a mouse model of MS, are major autoimmune demyelinating diseases of the central nervous system (CNS) (1, 2). Various types of immune cells and soluble mediators contribute to the complex mechanisms underlying the onset and progression of both MS and EAE, and recent studies have shown that type 1 helper T (Th1) cells and interleukin-17-producing helper T (Th17) cells play pivotal roles in their pathogenesis (3–5). In these diseases, autoreactive Th17 cells primed in the lymph nodes infiltrate the CNS and activate microglia/macrophages that induce inflammatory demyelination and subsequent neuronal damage, resulting in a wide range of clinical features, including sensory and motor paralysis, blindness, pain, incontinence, and dementia (1, 2).

Interleukin-19 (IL-19) is an IL-10 family cytokine that is homologous and highly similar to IL-20 and IL-24 (6, 7). IL-19 binds to the heterodimeric receptor consisting of IL-20Rα and IL-20Rβ, and its downstream signaling is mediated by STAT3 phosphorylation (8, 9). IL-19 is mainly produced by activated macrophages and microglia (10–13). Recent studies showed that IL-19 exerts anti-inflammatory effects on macrophages by inhibiting inflammatory cytokine production, downregulating antigen-presenting capacity, and enhancing M2 phenotype polarization, which promotes type 2 helper T (Th2) cell differentiation and suppresses Th1 and Th17 cell differentiation (11, 12, 14–17). In fact, IL-19 plays a critical role in development of various autoimmune diseases, including asthma (18, 19), psoriasis (20–22), inflammatory bowel disease (11, 23), rheumatoid arthritis (24), and Type I diabetes (25). However, it remains to be elucidated how IL-19 contributes to MS pathogenesis.

Here, we examined the pathological role of IL-19 in EAE using IL-19-deficient (IL-19^−/−^) mice. IL-19 deficiency markedly exacerbated EAE, and treatment with IL-19 effectively suppressed EAE accompanied by inhibiting macrophage antigen presentation and subsequent expansion of Th17 cells. Our findings suggest that IL-19 may provide significant therapeutic benefits for treating MS.

## Materials and Methods

### Reagents

MOG peptide 35–55 (MOG_35−55_; MEVGWYRSPFSRVVHLYRNGK) was synthesized and purified by Operon Biotechnologies (Tokyo, Japan). Incomplete Freund's adjuvant was obtained from Sigma-Aldrich (St. Louis, MO, USA). Heat-killed *Mycobacterium tuberculosis* H37Ra was obtained from Difco (Detroit, MI, USA), and pertussis toxin was obtained from List Biological Laboratories (Campbell, CA, USA). Recombinant mouse IL-6, IL-19, and TGF-β1 were obtained from R&D Systems (Minneapolis, MN, USA).

### Animals

All animal experiments were conducted under protocols approved by the Animal Experiment Committee of Nagoya University (approved numbers: 15017 and 15018) and Yokohama City University (approved number: F-A-19-036). C57BL/6J (B6) mice were purchased from Japan SLC (Hamamatsu, Japan). IL-19^−/−^ mice (B6 background) (11, 12) were obtained from Regeneron Pharmaceuticals (Tarrytown, NY, USA).

### EAE Induction and Treatment Studies

MOG-EAE was induced as previously described (26–28). In brief, 8-week-old female mice were immunized subcutaneously at the base of the tail with 0.2 ml of emulsion containing 200 μg MOG_35−55_ in saline, combined with an equal volume of complete Freund's adjuvant containing 300 μg heat-killed *Mycobacterium tuberculosis* H37Ra. The mice were intraperitoneally injected with 200 ng pertussis toxin on days 0 and 2 post-immunization. To investigate the effect of IL-19, EAE mice were treated with mouse recombinant IL-19 protein (20 ng/g of body weight) by intraperitoneal injection every other day starting on day 2 post-immunization, according to a modification of a previously reported method (29, 30). The mice were assessed daily for clinical signs of EAE, according to the following grading system: 0, normal; 1, limp tail or mild hind limb weakness; 2, moderate hind limb weakness or mild ataxia; 3, moderate to severe hind limb weakness; 4, severe hind limb weakness, mild forelimb weakness or moderate ataxia; 5, paraplegia with moderate forelimb weakness; and 6, paraplegia with severe forelimb weakness, severe ataxia, or moribundity.

### Isolation of Cells From Spleen and Lumbar Spinal Cord

Mononuclear cells were collected from the spleen and lumbar spinal cord as described previously (26–28). CD4^+^, CD11b^+^, and CD11c^+^ cells were isolated using the MACS system (Miltenyi Biotec, Bergisch Gladbach, Germany). Helper T cell differentiation was induced as described previously (5, 28, 31). For flow cytometric analysis, cells were stained with PerCP/Cy5.5 or BV421-conjugated anti-mouse CD4 rat monoclonal antibody (RM4-5; BD Biosciences, Franklin Lakes, NJ, USA). The cells were then fixed and permeabilized with Cytofix/Cytoperm reagent (BD Biosciences) and stained with PE-conjugated anti-mouse IFN-γ rat monoclonal antibody (XMG1.2; BD Biosciences) and APC- or PE-conjugated anti-mouse IL-17A rat monoclonal antibody (TC11-18H10; BD Biosciences). The samples were analyzed using a FACS Aria III system (BD Biosciences) and the FlowJo software (FlowJo, Ashland, OR, USA).

### Histological Analysis

Histological analysis was performed as previously described (26, 27). Mice with peak EAE were anesthetized and perfused transcardially with 4% paraformaldehyde in 0.1 M PBS. Lumbosacral spinal cords were immediately removed, post-fixed in 4% paraformaldehyde, and embedded in paraffin or OCT compound (Sakura Finetek Japan, Tokyo, Japan). Eight-micron-thick paraffin sections were stained with hematoxylin and eosin. Stained sections were analyzed on a NanoZoomer 2.0-RS slide scanner (Hamamatsu Photonics, Hamamatsu, Japan). For immunofluorescence staining, the frozen sections (20 μm) were permeabilized with 0.1% Triton X-100 in PBS for 20 min, blocked with 5% bovine serum for 30 min, and then incubated overnight with rat anti-mouse F4/80 monoclonal antibody (CI:A3-1; 1:1,000, AbD Serotec, Raleigh, NC, USA), rabbit anti-mouse iNOS polyclonal antibodies (1:5,000, Merck Millipore), and goat anti-mouse Arginase-1 polyclonal antibodies (1:200, Proteintech) followed by incubation with Alexa Fluor 488 or 546-conjugated secondary antibodies (Life Technologies, Carlsbad, CA, USA). The stained cells were analyzed by examining six random fields per section using a deconvolution fluorescence microscope system (BZ-X800, Keyence, Osaka, Japan). The data were collected from three animals per group.

### RNA Extraction and Reverse-Transcription Polymerase Chain Reactions (Rt-PCRs)

We evaluated the expression levels of proinflammatory factors in the lumbar spinal cords and spleens by qPCR as described previously (12, 26). In brief, lumbar spinal cords and spleens were collected from EAE mice at pre-immunization, EAE onset, and EAE peak (approximately on days 0, 10, 16 post-immunization, respectively). Total RNA was isolated with an RNeasy Mini Kit (Qiagen, Valencia, CA, USA) and reverse transcribed with SuperScript III (Life Technologies, Carlsbad, CA, USA). Expression levels of mRNAs were evaluated by qPCR using SYBR Select Master Mix (Applied Biosystems, Foster City, CA, USA) on a Rotor-Gene Q (Qiagen) or LightCycler 96 (Roche). Mouse gene-specific primers were obtained from Life Technologies (Table 1). Gene-expression values were determined using the ΔΔC_T_ method. Levels of mRNAs of interest were standardized to the geometric mean of the level of hypoxanthine phosphoribosyltransferase 1 (*Hprt1*). Assays were carried out in three independent trials.

**Table 1:** Primers for qPCR.

**Gene**	**Primer sequence**
mouse *Il19* sense	CAACCTGCTGACATTCTACAGAG
mouse *Il19* antisense	CCTGACATCGCTCCAGAGATTT
mouse *Il17a* sense	TCATCTGTGTCTCTGATGCTGTTG
mouse *Il17a* antisense	TCGCTGCCTTCACTGT
mouse *Ifng* sense	TGGCATAGATGTGGAAGAAAAGAG
mouse *Ifng* antisense	TGCAGGATTTTCATGTCACCAT
mouse *Foxp3* sense	TTCATGCATCAGCTCTCCAC
mouse *Foxp3* antisense	CTGGACACCCATTCCAGACT
mouse *Il1b* sense	GAAATGCCACCTTTTGACAGTG
mouse *Il1b* antisense	TGGATGCTCTCATCAGGACAG
mouse *Il6* sense	TCTATACCACTTCACAAGTCGGA
mouse *Il6* antisense	GAATTGCCATTGCACAACTCTTT
mouse *Tgfb1* sense	CGAAGCGGACTACTATGCTAAAGA
mouse *Tgfb1* antisense	GTTTTCTCATAGATGGCGTTGTTG
mouse *Il10* sense	GAGAAGCATGGCCCAGAAATC
mouse *Il10* antisense	CGCATCCTGAGGGTCTTCA
mouse *Il12p40* sense	GGTGCAAAGAAACATGGACTTG
mouse *Il12p40* antisense	CACATGTCACTGCCCGAGAGT
mouse *Il23p19* sense	GCACCAGCGGGACATATGA
mouse *Il23p19* antisense	CCTTGTGGGTCACAACCATCT
mouse *Tnfa* sense	GACCCTCACACTCAGATCATCTTCT
mouse *Tnfa* antisense	CCACTTGGTGGTTTGCTACGA
mouse *Il20ra* sense	GGAAACTCAAGTCAGCCCAC
mouse *Il20ra* antisense	AGATGGACTTCTCGCCAGTT
mouse *Il20rb* sense	CCGAAATGCAACTGTCCTCAC
mouse *Il20rb* antisense	AATAACCAGATGCAGCCCATGT
mouse *Rorgt* sense	GCGACTGGAGGACCTTCTAC
mouse *Rorgt* antisense	TCCCACATTGACTTCCTCTG
mouse *H2ab1* sense	AGACGCCGAGTACTGGAACAGCCAGC
mouse *H2ab1* sense	CAGAGTGTTGTGGTGGTTGAGGGCCTC
mouse *Cd80* sense	CATCAAAGCTGACTTCTCTACCC
mouse *Cd80* antisense	GGGTTTTTCCCAGGTGAAGT
mouse *Cd86* sense	TCAGTGATCGCCAACTTCAG
mouse *Cd86* sense	GAAACTCTTGAGTGAAATTGAGAGG
mouse *Hprt1* sense	CAGTCAACGGGGACATAAA
mouse *Hprt1* antisense	GGGGCTGTACTGCTTAACCAG

### Statistical Analysis

Statistical significance was analyzed using Student's *t*-test, one-way analysis of variance (ANOVA), or repeated measures ANOVA followed by *post-hoc* Tukey's test in GraphPad Prism version 8 (GraphPad Software, La Jolla, CA, USA).

## Results

### IL-19 Deficiency Exacerbates EAE

**Figure 1:**
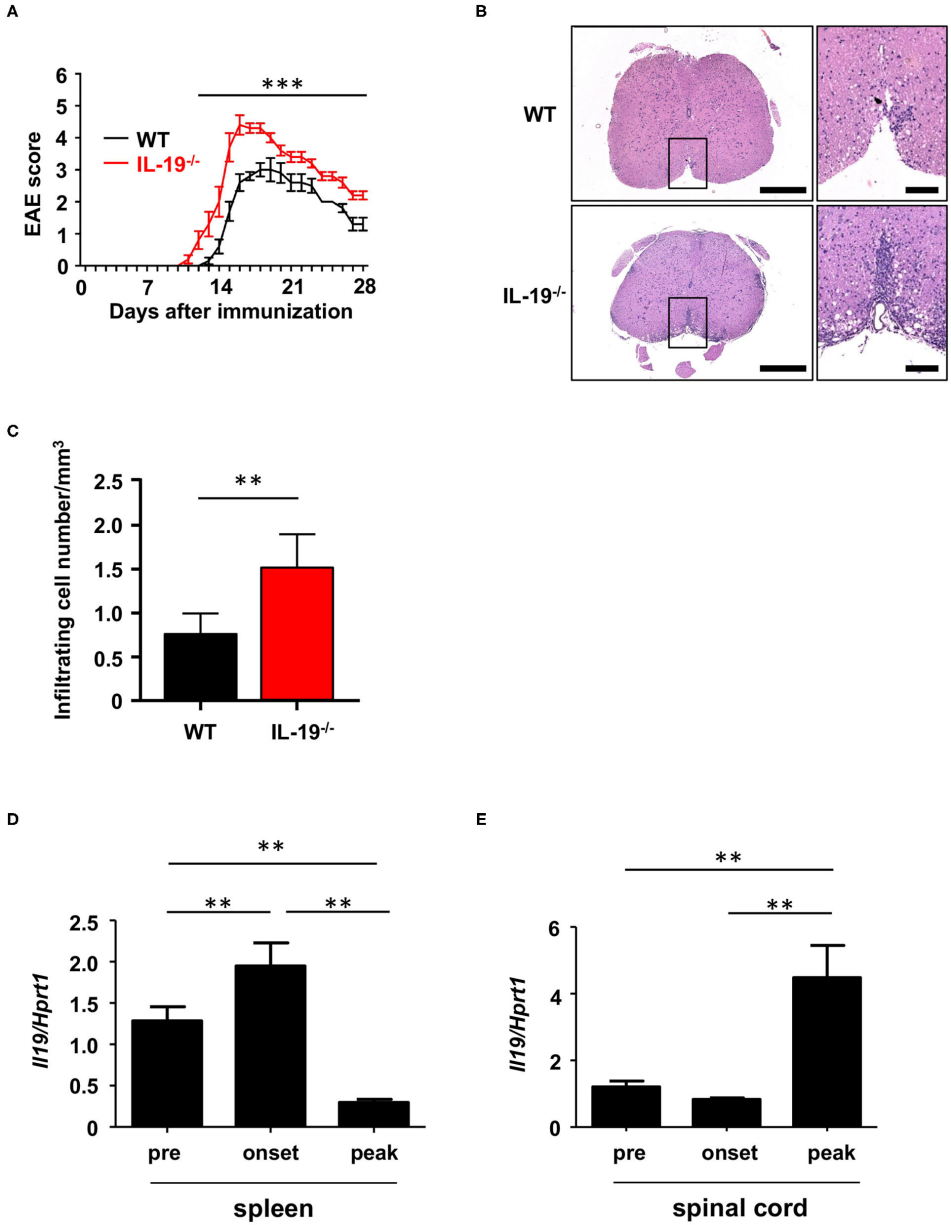
IL-19 deficiency aggravates EAE. **(A)** EAE clinical scores for WT (black) and IL-19^−/−^ (red) mice. Statistical significance was analyzed using repeated measures ANOVA followed by *post-hoc* Tukey's test. Data represent means ± SD (*n* = 10). ***p < 0.0001. **(B)** Micrographs of hematoxylin/eosin staining of L5 lumbar spinal cords at the peak EAE of WT and IL-19^−/−^ mice. The right panels show enlargements of the boxed areas in the left panels. Scale bars: 500 μm (left), 100 μm (right). **(C)** Quantitative analysis of cell infiltration in L5 lumbar spinal cords at the peak EAE (*n* = 5). Statistical significance was analyzed using Student's *t*-test. Data represent means ± SEM. **p < 0.01. **(D)** Chronological qPCR data for IL-19 mRNA expression level in the spleen of WT EAE mice (*n* = 5). **(E)** Chronological qPCR data for IL-19 mRNA expression level in the lumbar spinal cord of WT EAE mice (*n* = 5). Pre, pre-immunization; onset, EAE onset; peak, EAE peak. Statistical significance was analyzed using one-way ANOVA followed by *post-hoc* Tukey's test. Data represent means ± SD. **p < 0.01.

We generated myelin oligodendrocyte glycoprotein (MOG)-induced EAE in C57BL/6J wild-type (WT) and IL-19-deficient (IL-19^−/−^) mice. We already confirmed that IL-19^−/−^ mice without immunization showed no inflammatory cell infiltration in the CNS as well as WT mice (Supplementary Figure 1). IL-19^−/−^ mice exhibited earlier disease onset and more severe symptoms than WT mice (Figure 1A). Histological analysis of the lumbar spinal cords revealed more inflammatory cell infiltration in IL-19^−/−^ mice than in WT mice (Figure 1B). Quantitative analysis also disclosed that IL-19^−/−^ EAE mice had more infiltrating cells in the lumbar spinal cords than WT EAE mice (Figure 1C). We then chronologically evaluated IL-19 expression levels in the spleen and lumbar spinal cord of WT mice at pre-immunization, disease onset, and disease peak. Splenic IL-19 mRNA expression was upregulated at disease onset, but was strongly suppressed at the disease peak (Figure 1D). By contrast, upregulation of IL-19 mRNA in the lumbar spinal cord was observed at disease peak (Figure 1E). These results suggest that endogenous IL-19 serves as a negative regulator of EAE pathogenesis at both the induction and effector phases.

### IL-19 Deficiency Increases Th17 Cell Infiltration Into the CNS

**Figure 2:**
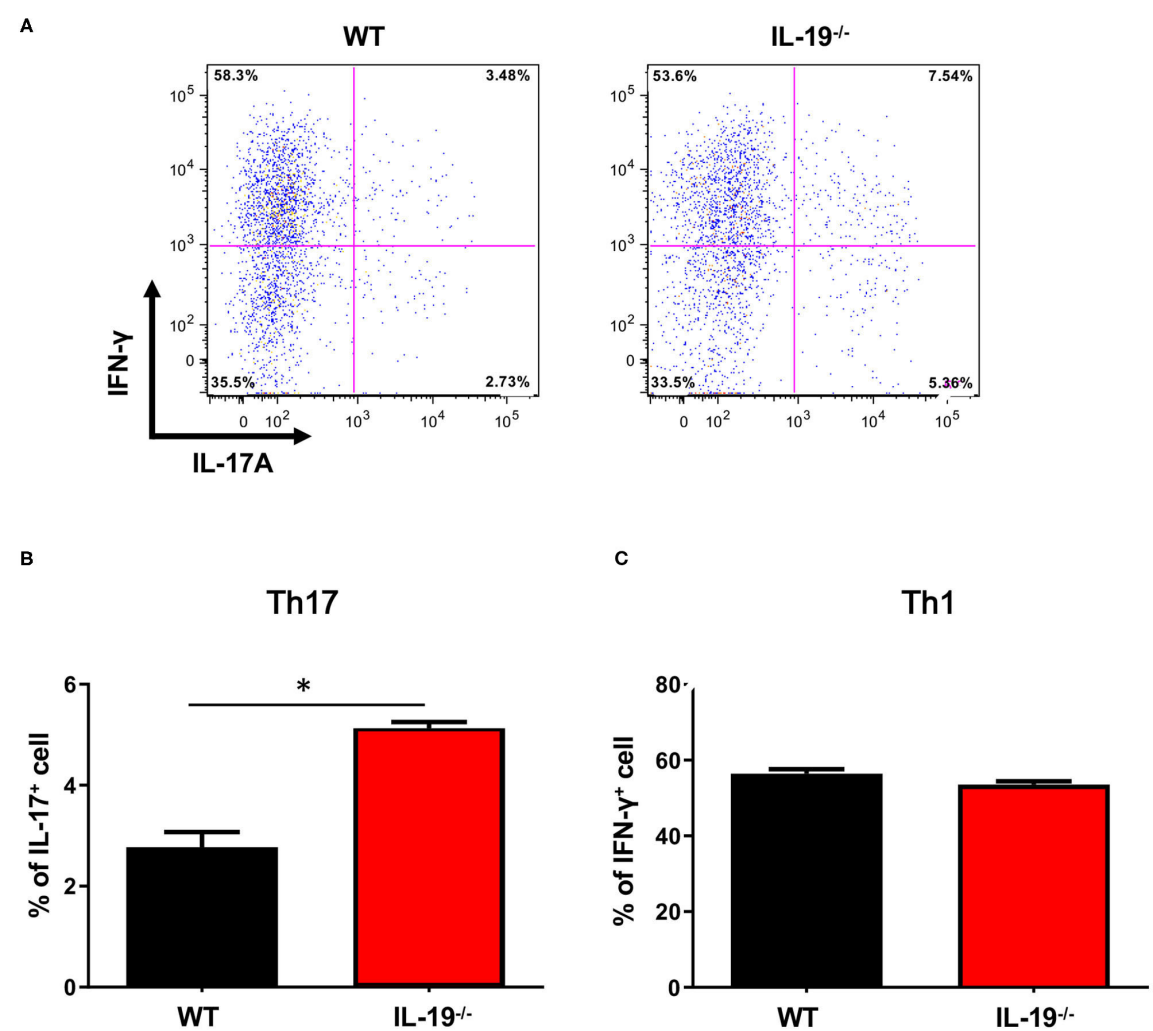
IL-19 deficiency increases CNS infiltration of Th17 cells, but not Th1 cells. **(A)** Representative flow-cytometric data for IL-17- and IFN-γ-producing CD4^+^ T cells in the CNS at the peak EAE. **(B)** Percentage of IL-17-producing CD4^+^ T cells. **(C)** Percentage of IFN-γ-producing CD4^+^ T cells. Statistical significance was analyzed using Student's *t*-test. Data represent means ± SEM (*n* = 3). *p < 0.05.

Because EAE is a Th1 and Th17 cell-mediated autoimmune disease, we next assessed whether IL-19 deficiency would increase CNS infiltration of Th1 and Th17 cells. Flow cytometric analysis revealed that at disease peak, IL-19^−/−^ mice exhibited more infiltration of Th17 cells in the spinal cord than WT mice (Figures 2A,B). By contrast, no difference was observed in Th1 cell infiltration between IL-19^−/−^ and WT mice (Figure 2C). These results indicate that IL-19 deficiency mediates elevated CNS infiltration by Th17 cells, but not Th1 cells.

### IL-19 Deficiency Expands Th17 Cell Population

**Figure 3:**
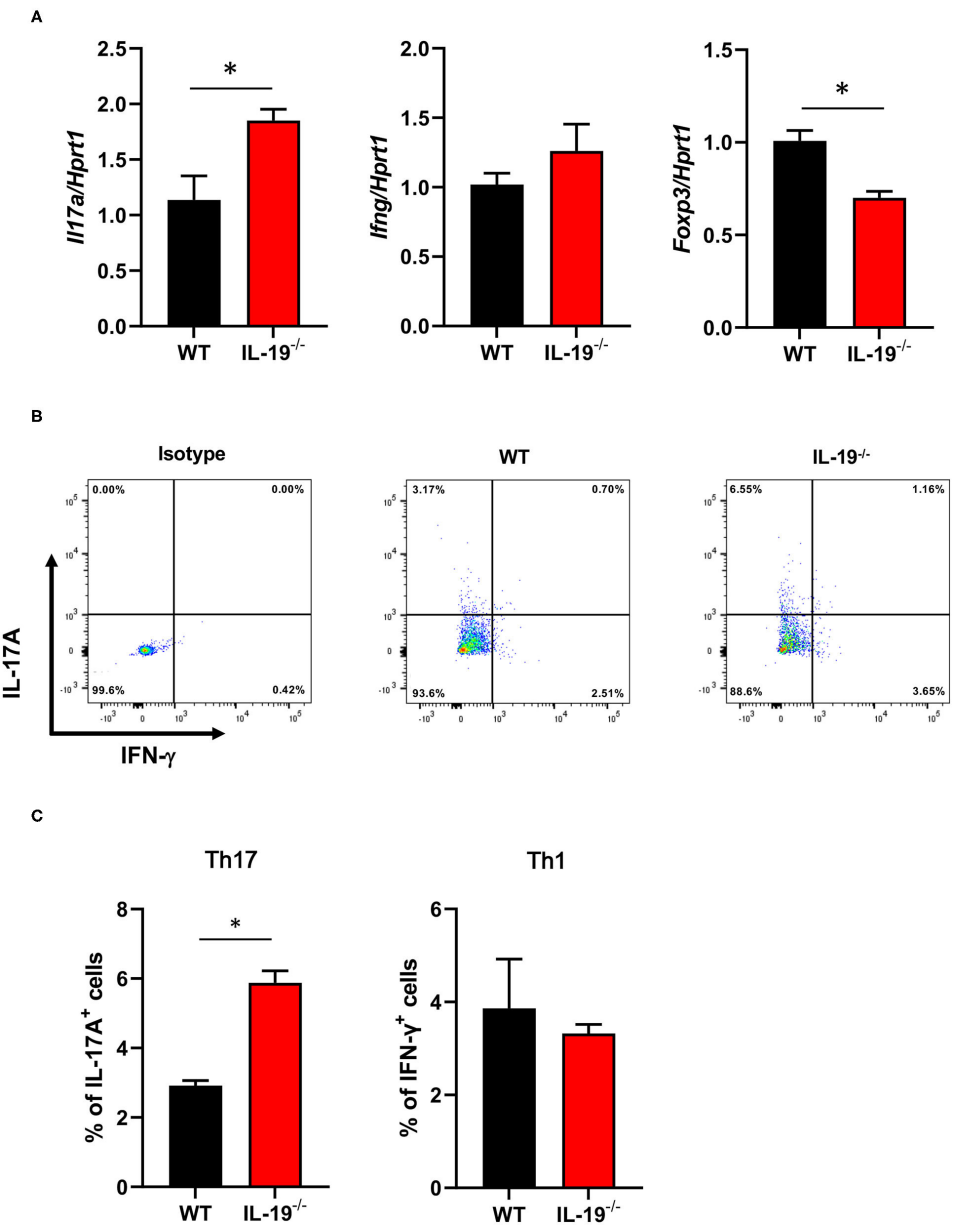
IL-19 deficiency expands the Th17 cell population. **(A)** qPCR data for levels of mRNAs encoding IL-17A, IFN-γ, and FoxP3 in splenic CD4^+^ T cells **(B)** Representative flow cytometric data for IL-17 and IFN-γ-producing CD4^+^ T cells in the spleens of EAE mice. **(C)** Percentage of IL-17 and IFN-γ-producing CD4+ T cells in the spleens of EAE mice. Cells were isolated from WT and IL-19^−/−^ EAE mice on 7 days after immunization and stimulated with MOG peptide for 3 days *in vitro*. Statistical significance was analyzed using Student's *t*-test. Data represent means ± SEM (*n* = 3). *p < 0.05.

To determine whether IL-19 contributes to the expansion of Th17 cells during the induction phase of EAE, we assessed the antigen-specific expansion of Th17 cells *ex vivo*. Splenic CD4^+^ T cells isolated from WT and IL-19^−/−^ mice at MOG-EAE onset were stimulated with MOG peptide for 3 days *in vitro*. IL-19 deficiency significantly upregulated *Il17a* mRNA and downregulated *Foxp3* mRNA, but it did not affect the level of *Ifng* mRNA (Figure 3A). Flow cytometric analysis also revealed that IL-19 deficiency expanded the Th17 cell population (Figures 3B,C). These results indicate that IL-19 deficiency mediates expansion of Th17 cells in the peripheral lymphoid tissues.

### IL-19 Deficiency Skews Cytokine Expression Profiles Toward Th17 Cell Expansion in Macrophages

We then examined how IL-19 deficiency expands Th17 cells in the induction phase of EAE. First, we evaluated the mRNA expression level of IL-19 receptor (heterodimer of IL-20Rα and IL-20Rβ subunits) in the splenic immune cells such as macrophage, dendritic cell (DC), and CD4^+^ helper T cell. We found that both the IL-20Rα and IL-20Rβ subunits were more highly expressed in CD11b^+^ macrophages and CD4^+^ helper T cells than in CD11c^+^ DCs (Supplementary Figure 2). These results suggest that IL-19 mainly affects macrophages and CD4^+^ helper T cells.

Next, we assessed whether IL-19 directly differentiates naïve CD4^+^ T cells into Th17 cells. Naïve T cells were polarized using immobilized CD3 and CD28 antibodies in the presence of IL-6 and transforming growth factor β1 (TGF-β1), with or without IL-19. Quantitative PCR (qPCR) and flow cytometry revealed that IL-19 did not alter the differentiation of naïve T cells into Th17 cells (Supplementary Figure 3).

**Figure 4:**
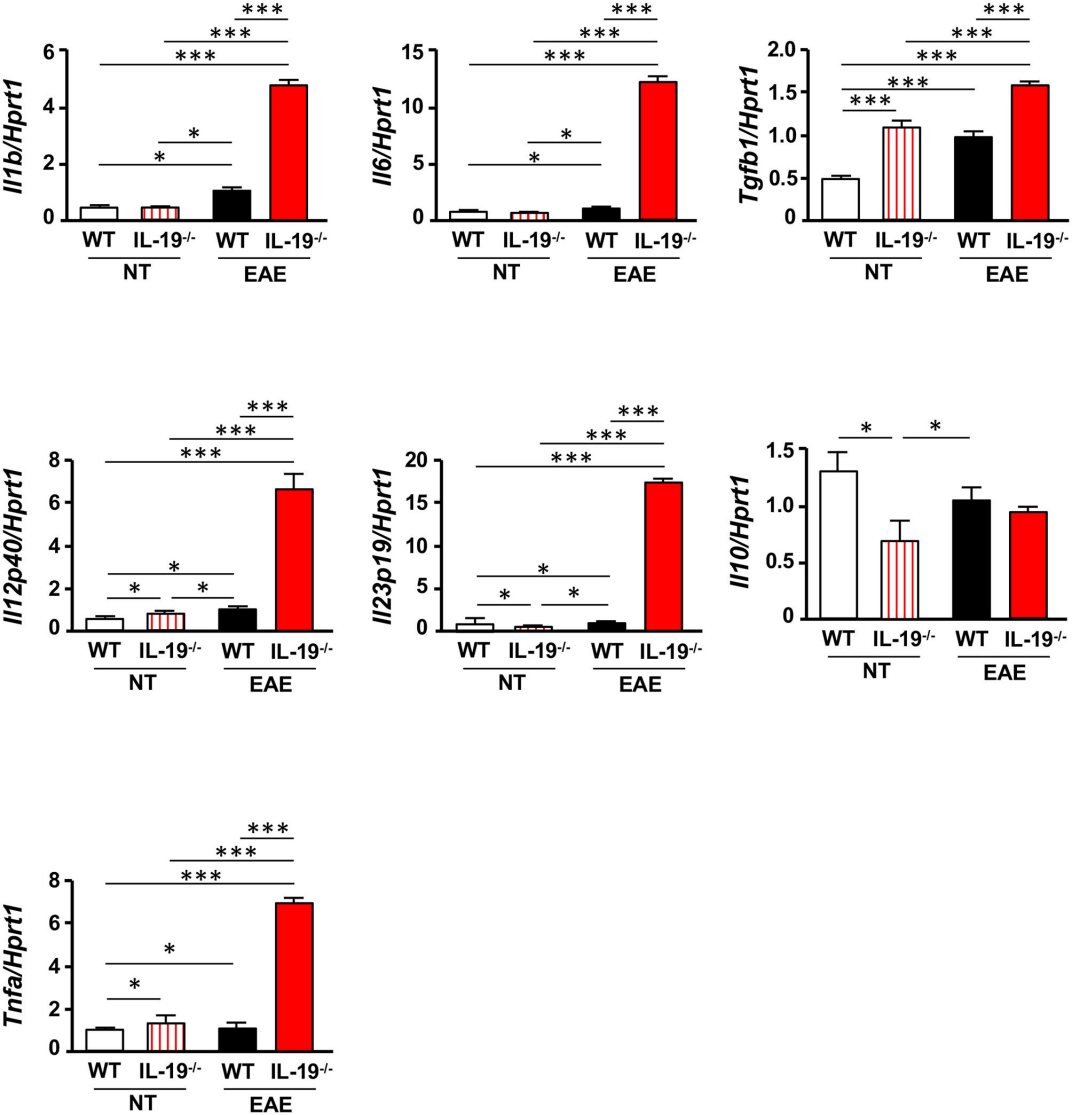
IL-19 deficiency upregulates Th17 cell differentiation–associated cytokines in macrophages. qPCR data for mRNAs encoding IL-1β, IL-6, TGF-β1, IL-12 p40, IL-23 p19, IL-10, and TNF-α in splenic macrophages of untreated (non-immunized) mice or EAE mice. Assessments of the samples from EAE mice were performed 7 days after immunization. Statistical significance was analyzed using one-way ANOVA followed by *post-hoc* Tukey's test. Data represent means ± SEM. *p < 0.05; ***p < 0.0001 (*n* = 6). NT, untreated mice.

Because antigen-presenting cells (APCs) are crucial for differentiation of naïve T cells into effector T cells, we evaluated the expression levels of cytokines required for Th17 cell expansion in splenic macrophages and DCs. Interestingly, IL-19^−/−^ macrophages from EAE mice exhibited a significant increase in mRNA levels of the genes encoding IL-1β, IL-6, TGF-β1, IL-12 p40, IL-23 p19, and tumor necrosis factor α (TNF-α), which play pivotal roles in Th17 cell differentiation and expansion (Figure 4). Although a previous study showed that IL-19 increases IL-10 expression (32), our data showed that IL-19 deficiency did not alter the *Il10* mRNA level in macrophages in EAE whereas IL-19 deficiency downregulated *Il10* mRNA expression in non-immunized macrophages (Figure 4). By contrast, IL-19^−/−^ DCs did not exhibit a significant alteration in the expression levels of these cytokines (Supplementary Figure 4). These findings indicated that IL-19 deficiency skews the cytokine expression profiles toward Th17 cell differentiation and expansion in macrophages. Conversely, our data suggested that IL-19 suppresses Th17-skewed condition by activating macrophages.

### IL-19 Deficiency Promotes MHC Class II Expression in Macrophages

**Figure 5:**
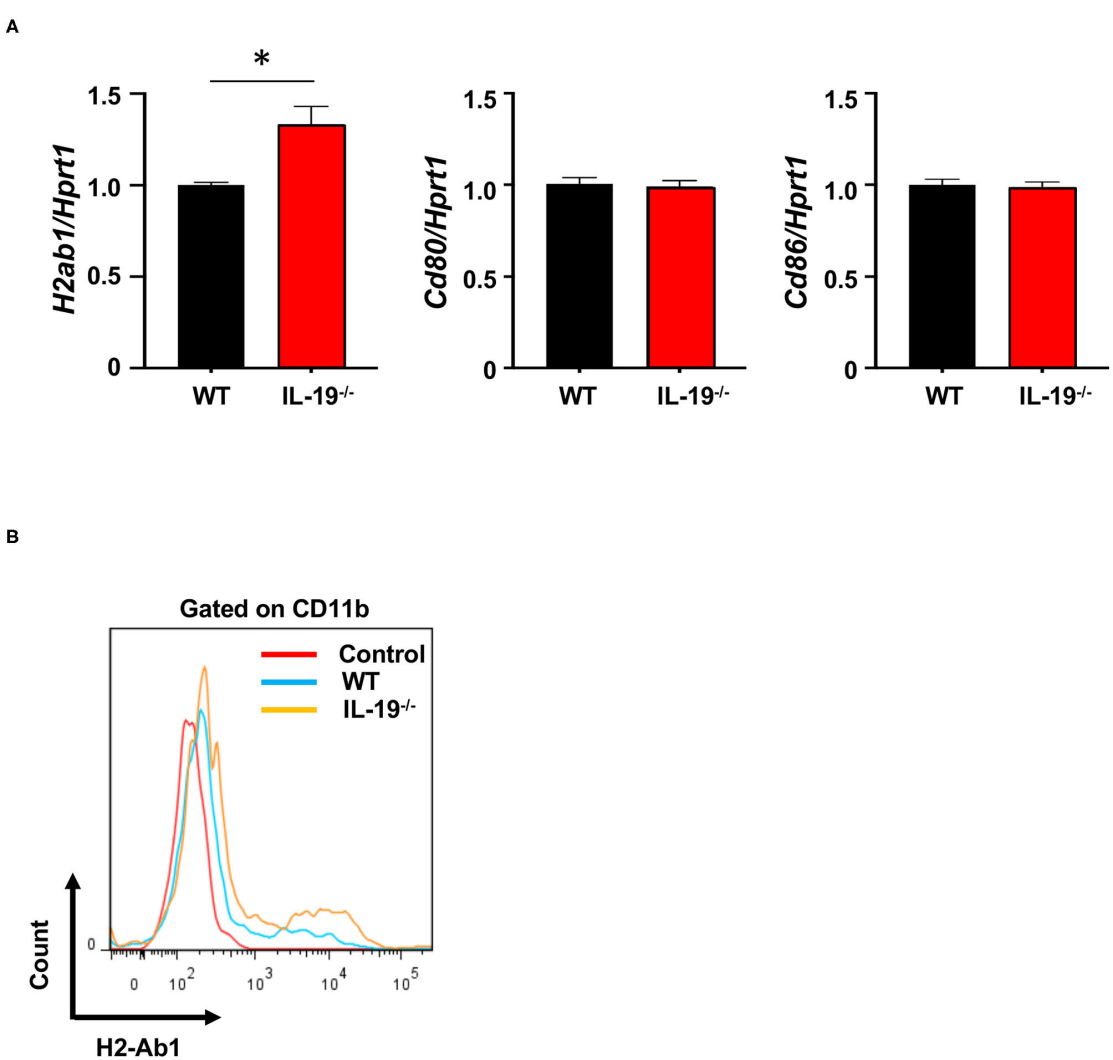
IL-19 deficiency enhances antigen-presenting activity in macrophages. **(A)** qPCR data for mRNAs encoding MHC class II (H2-Ab), CD80, and CD86 in the splenic macrophages on day 7 after immunization. Data represent means ± SD. *p < 0.05 (*n* = 6). **(B)** Representative flow cytometric data for MHC class II (H2-Ab) presentation in splenic macrophages.

To determine whether IL-19 signaling contributes to antigen presentation by macrophages, we assessed the expression of major histocompatibility complex (MHC) class II (H2-Ab) and co-stimulatory molecules (CD80 and CD86) in splenic CD11b^+^ macrophages from WT and IL-19^−/−^ EAE mice (Figure 5A). IL-19 deficiency significantly enhanced expression of the gene encoding MHC class II, whereas the genes encoding co-stimulatory molecules CD80 and CD86 were not affected (Figure 5A). Flow cytometric data corroborated the enhanced presentation of MHC class II in IL-19^−/−^ splenic macrophages (Figure 5B). These observations suggested that IL-19 also suppresses the antigen-presenting activity of macrophages.

### Treatment With IL-19 Abrogates EAE

**Figure 6:**
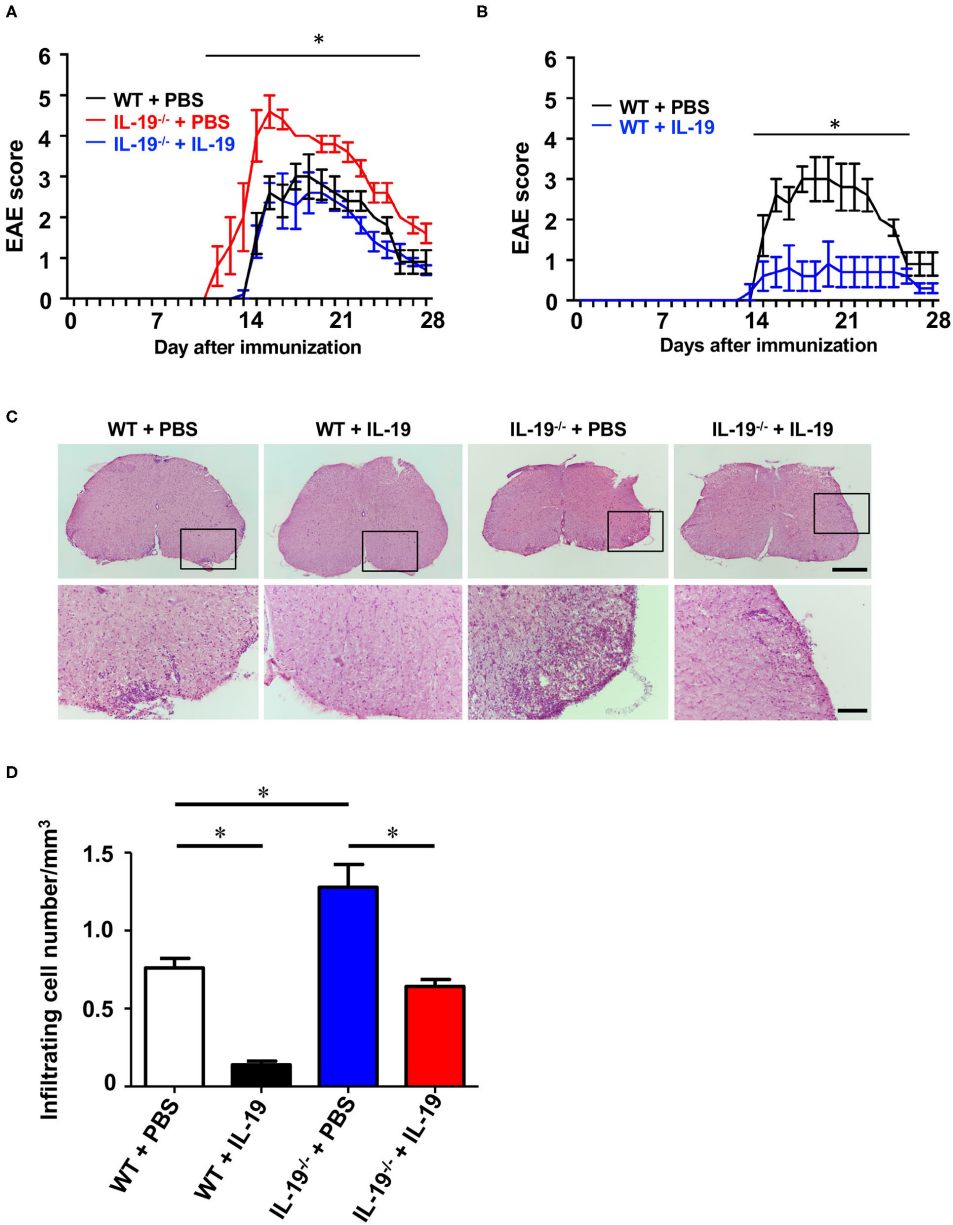
Treatment with recombinant IL-19 alleviates EAE. **(A)** EAE clinical score. WT + PBS (black): WT EAE mice treated with PBS; IL-19^−/−^ + PBS (red): IL-19^−/−^ EAE mic treated with PBS; IL-19^−/−^ + IL-19 (blue): IL-19^−/−^ EAE mice treated with IL-19. **(B)** EAE clinical score. WT + PBS (black), WT EAE micee treated with PBS; WT + IL-19 (blue), WT EAE mice treated with IL-19. Statistical significance was analyzed using repeated measures ANOVA followed by *post-hoc* Tukey's test. Data represent means ± SD. *p < 0.05 (*n* = 5). **(C)** Micrographs of hematoxylin/eosin staining of L5 lumbar spinal cords of EAE mice. The bottom panels show enlargements of the boxed areas in the upper panels. Scale bars: 500 μm (upper), 100 μm (bottom). **(D)** Quantitative analysis of cell infiltration in L5 lumbar spinal cords at the peak EAE (*n* = 5). Statistical significance was analyzed using repeated one-way ANOVA followed by *post-hoc* Tukey's test. Data represent means ± SEM. *p < 0.05.

Then, we examined whether exogenous IL-19 abolishes the effect of IL-19 deficiency in EAE. According to the previous studies showing that the daily administration of IL-19 protein (10 ng/g of body weight) can offset IL-19 deficiency (29, 30), we firstly treated WT and IL-19^−/−^ EAE mice with recombinant mouse IL-19 protein (10 ng/g of body weight) or PBS by intraperitoneal injection every day. However, even daily injection with PBS caused mice severe stress which markedly suppressed EAE development (33). Therefore, we decided to treat mice every other day because this frequency of treatment did not cause mice enough stress to suppress EAE. We treated IL-19^−/−^ EAE mice with recombinant mouse IL-19 protein (20 ng/g of body weight) by intraperitoneal injection every other day, starting on day 2 post-immunization. As expected, administration of IL-19 to IL-19^−/−^ mice improved EAE to the similar level to WT EAE mice, indicating that IL-19 treatment completely offset the effect of IL-19 deficiency (Figure 6A, IL-19^−/−^ + IL-19). Limitations of solubility and injection volume did not allow us to increase the dosage. We then investigated the therapeutic effect of IL-19 on EAE. When we treated WT EAE mice with recombinant mouse IL-19 protein in the same manner (i.e., their IL-19 level were estimated twice as much as the baseline level in untreated WT mice), we found that IL-19 treatment almost completely inhibited EAE (Figure 6B). Histological analysis revealed that the clinical improvement accorded with the level of inflammatory cell infiltration in the lumbar spinal cords (Figures 6C,D). These results indicated that IL-19 represents a potential target for MS therapy.

### Treatment With IL-19 Did Not Alter Macrophage Phenotype

**Figure 7:**
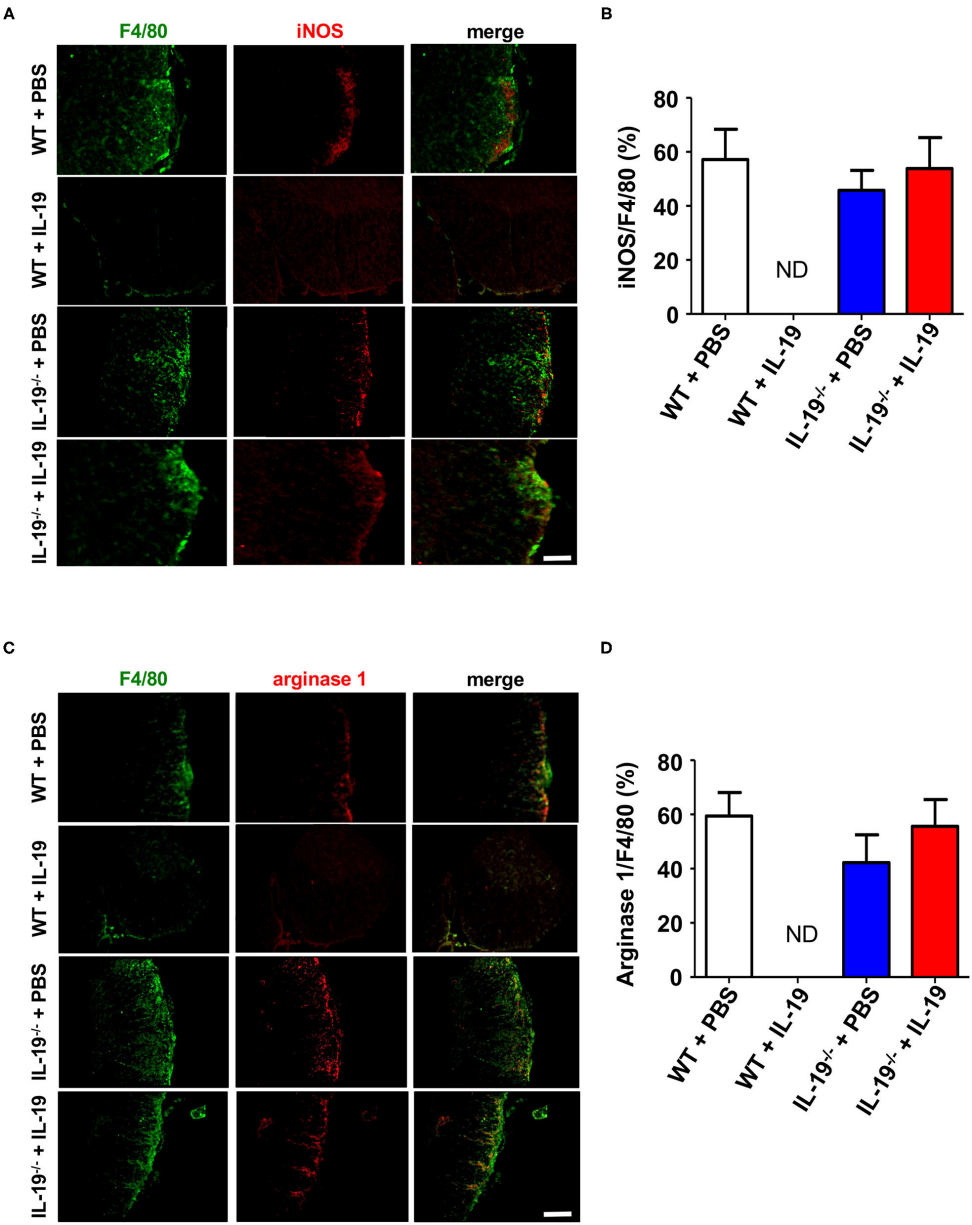
Treatment with recombinant IL-19 did not alter macrophage phenotype. **(A)** Representative immunohistological images of L5 lumbar spinal cord sections from EAE mice. Pro-inflammatory M1 macrophage (iNOS) were examined. **(B)** Quantitative data of A. IL-19 treatment did not alter the number of iNOS^+^ F4/80^+^ M1 cells albeit F4/80^+^ cells were not detected in IL-19–treated WT EAE mice. **(C)** Representative immunohistological images of L5 lumbar spinal cord sections from EAE mice. Anti-inflammatory M2 macrophage (arginase 1) were examined. **(D)** Quantitative data of **(C)**. IL-19 treatment did not alter M1/M2 cell population although F4/80^+^ cells were not detected in IL-19–treated WT EAE mice. Scale bars, 100 μm. Statistical significance was analyzed using repeated measures ANOVA followed by *post-hoc* Tukey's test. Data represent means ± SEM (*n* = 5). ND, not detected.

To determine whether IL-19 treatment converts macrophage phenotype, we assessed macrophage M1/M2 phenotype by immunofluorescent staining for iNOS (M1 marker) and arginase 1 (M2 marker) in the infiltrating macrophage (F4/80^+^). Immunostaining data revealed that IL-19 treatment did not alter M1/M2 macrophage population although infiltrating F4/80^+^ macrophage were not detected in IL-19-treated WT EAE mice (Figure 7). These observations suggested that main therapeutic effect of IL-19 on EAE is suppression of inflammatory cell infiltration in the CNS, but not M1/M2 phenotype conversion.

## Discussion

IL-19 has been considered to be a Th2 cytokine that promotes Th2-skewed diseases such as asthma, atopic dermatitis, psoriasis, and rheumatoid arthritis (34), however, IL-19 often exerts controversial effects depending on species and types of immune cells (35). In inflammatory bowel disease, IL-19 exerts anti-inflammatory effect to hamper hyperactivation of both innate and acquired immunity (11, 23). As to asthma, in human and murine models, IL-19 exacerbates the disease by a positive-feedback on Th2 cells and M2 macrophage via upregulation of such Th2 cytokines as IL-4, IL-5, IL-10, and IL-13 (18, 19, 32, 36), whereas IL-19 also promotes M1 macrophage by upregulation of such Th1 cytokines as IL-6 and TNF-α (37). Although this dichotomy in response to IL-19 may partially derive from the differences in phenotype of mice used (i.e., Th1-skewed C57BL/6 mice vs. Th2-skewed BALB/c mice), a growing evidence suggests that IL-19 exerts pleiotropic roles in both pro- and anti-inflammatory responses among different types of immune cells.

In this study, we demonstrated that endogenous IL-19 negatively regulates development of EAE by inhibiting macrophage activation, and that IL-19 treatment effectively abrogates EAE. As shown in Figures 1D,E, endogenous *Il19* mRNA expression was upregulated at EAE onset and downregulated at EAE peak in the spleen, whereas it was elevated at EAE peak in the CNS. We have previously identified IL-19 as a negative-feedback regulator to limit proinflammatory response of macrophages and microglia in autocrine/paracrine manners (11, 12). From this point of view, these data imply that endogenous IL-19 increases to suppress inflammation accompanied by macrophage/microglia activation as disease progresses from the periphery to CNS, although it is insufficient to halt EAE progression. It is also intriguing that splenic *Il19* mRNA expression decreased at EAE peak much less than at pre-immunization. A previous study showed that IL-19 increases IL-10 which downregulates IL-19, suggesting that IL-10 is a negative-feedback regulator of IL-19 (32). In accordance with this fact, our qPCR data also showed that IL-19 deficiency downregulated *Il10* mRNA expression (Figure 4). Since IL-10 increases during recovery phase of EAE (38), it is plausible that IL-19-induced IL-10 downregulates splenic IL-19 at the peak EAE. Accordingly, axis of IL-19 and IL-10 may be a critical inducer of remission in MS. In fact, a recent study disclosed that serum IL-19 levels were lower in the relapse phase than the remission phase in patients with neuromyelitis optica (39).

Th17 cell infiltration in the CNS is considered critical for the development of EAE (5, 40), and APCs such as macrophages, microglia, and DC are also essential for effector T cell differentiation and expansion in EAE (41). Specifically, IL-1β, IL-6, IL-23, TGF-β1, and TNF-α released by APCs play pivotal roles in Th17 cell differentiation and expansion (5, 31, 42–45). In this study, we revealed that IL-19 deficiency significantly upregulated mRNA levels of these Th17 cell differentiation-associated cytokines in macrophages, but not in DCs (Figure 4 and Supplementary Figure 3), although these protein levels have not been evaluated in this study. These phenomena were correlated with the expression level of IL-19 receptor (IL-20Rα and IL-20Rβ heterodimer), which was highly expressed in macrophages, but not in DCs or helper T cells (Supplementary Figure 1). IL-19 deficiency further enhanced MHC class II expression in macrophages, which enable to prime T cells (46). Taken together, our findings suggest that IL-19 suppresses Th17 cell differentiation and expansion by suppressing cytokine production and antigen presentation in macrophages. Interestingly, a previous study reported that IL-17A induces IL-19 production (19). Thus, IL-19 also might serve as a negative regulator of further Th17 cell polarization.

In addition, activated macrophages and microglia directly contribute to neuroinflammation-induced demyelination by releasing proinflammatory cytokines such as IL-1β, IL-6, and TNF-α (41, 47–49). In our study, IL-19 deficiency increased the levels of these proinflammatory cytokines in macrophages and exacerbated EAE until the late phase of the disease. We previously revealed that IL-19 secreted from activated macrophages and microglia suppressed their proinflammatory responses in an autocrine/paracrine manner (11, 12). Therefore, IL-19 might suppress development of EAE by dual inhibition of both autoreactive Th17 cell expansion and macrophage/microglia-mediated CNS neuroinflammation.

While IL-19 shares its receptor IL-20Rα and IL-20Rβ with IL-20 and IL-24, IL-20 and IL-24 also bind another heterodimer receptor IL-22Rα1 and IL-20Rβ (50). Therefore, these cytokines have unique biological functions as well as common functions including promoting Th2-skew inflammation. For example, unlike IL-19, IL-20 promotes DC cell maturation (51) and IL-24 exerts antitumorigenic effects (52). It is still uncertain whether these unique functions depend on the restricted expression pattern of their receptors or the synergistic effects of downstream signaling pathways (50). Although the IL-19 signaling pathway has not been fully elucidated, IL-19 mediates its downstream signaling at least by STAT3 activation (8, 9, 12, 53). However, it remains controversial whether STAT3 activation is beneficial or harmful with regard to autoimmune-mediated neuroinflammation. Previous studies also reported that STAT3 activation in myeloid cells (including macrophages and microglia) exacerbates EAE (54, 55). By contrast, STAT3 ablation worsens neuroinflammation in mice with spinal cord injury (56), and STAT3 activation alleviates cuprizone-induced CNS demyelination (57). This discordancy may depend on spatiotemporally specific activation of STAT3 (58). Indeed, in contrast to macrophages, IL-19 deficiency did not affect activation of DCs. Recent clinical trials of JAK/STAT inhibitors for autoimmune diseases have revealed a complicated signal network of cytokine/STAT axes in multiple cell types (59). Further studies are needed to elucidate the precise IL-19 signaling pathway in each cell type.

## Conclusions

In this study, we revealed that IL-19 deficiency exacerbated EAE by upregulating Th17 cell differentiation-associated cytokines and enhancing antigen presentation in macrophages, followed by Th17 cell expansion and infiltration in the CNS. We also demonstrated that IL-19 administration potently prevented development of EAE. Therefore, enhancement of IL-19 signaling represents a promising therapeutic strategy against MS and other Th17-mediated autoimmune diseases.

## Data Availability Statement

The raw data supporting the conclusions of this article will be made available by the authors, without undue reservation.

## Ethics Statement

The animal study was reviewed and approved by Animal Experiment Committee of Nagoya University and Yokohama City University.

## Author Contributions

HH, BP, AS, and HT designed the research. HH, BP, HK, YO, SJ, KT, Y-TA, and HT performed the research. HH, BP, HK, FT, AS, and HT analyzed the data. and HH, BP, and HT wrote the paper. All authors contributed to the article and approved the submitted version.

## Conflict of Interest

The authors declare that the research was conducted in the absence of any commercial or financial relationships that could be construed as a potential conflict of interest.
