# Supplementary figures and images for: Interleukin-19 Abrogates Experimental Autoimmune Encephalomyelitis by Attenuating Antigen-Presenting Cell Activation
Source: Front Immunol. 2021 Mar 11;12:615898. doi: 10.3389/fimmu.2021.615898 (PMC7990911; doi:10.3389/fimmu.2021.615898)

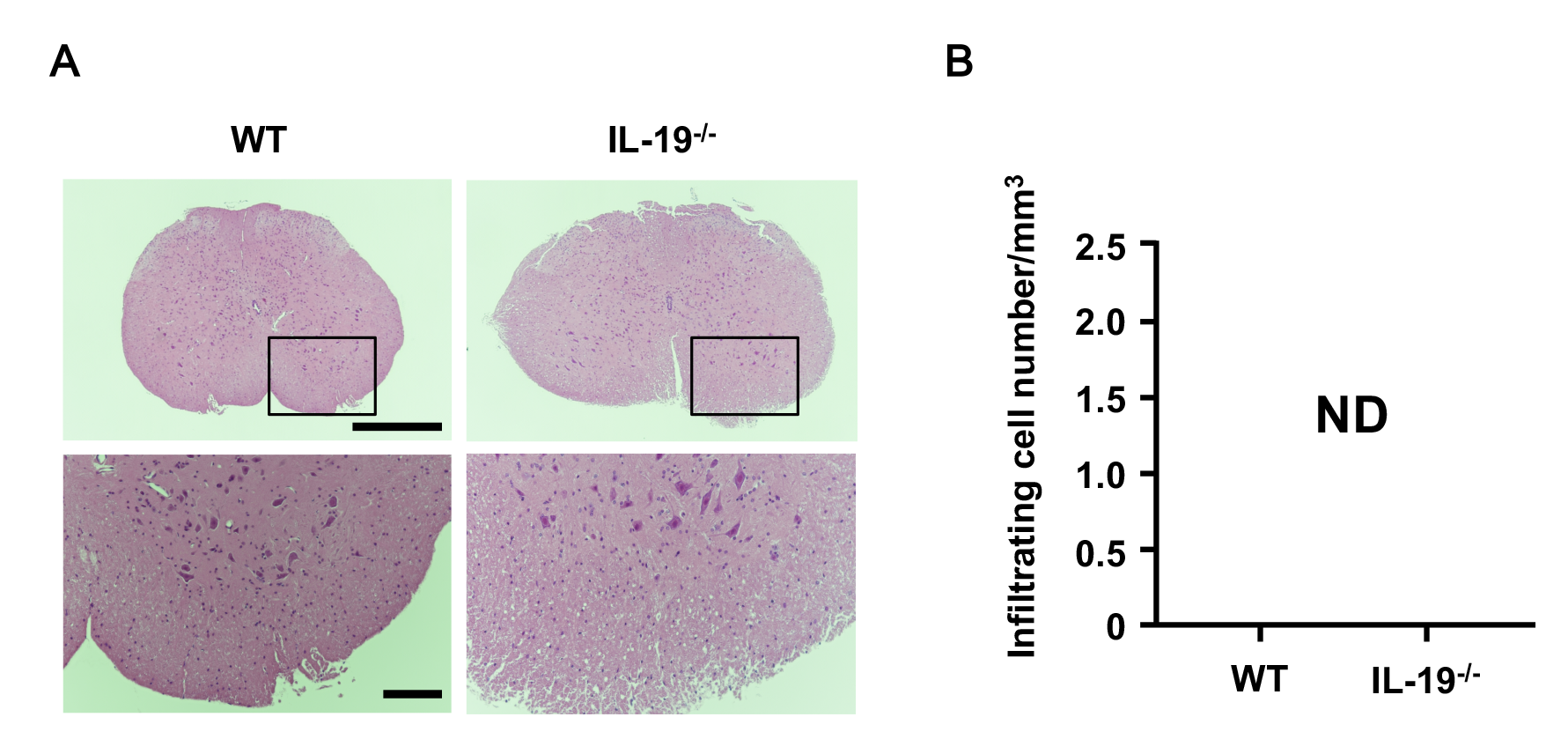

Supplement: Supplementary file 1 [file Image_1.TIF]

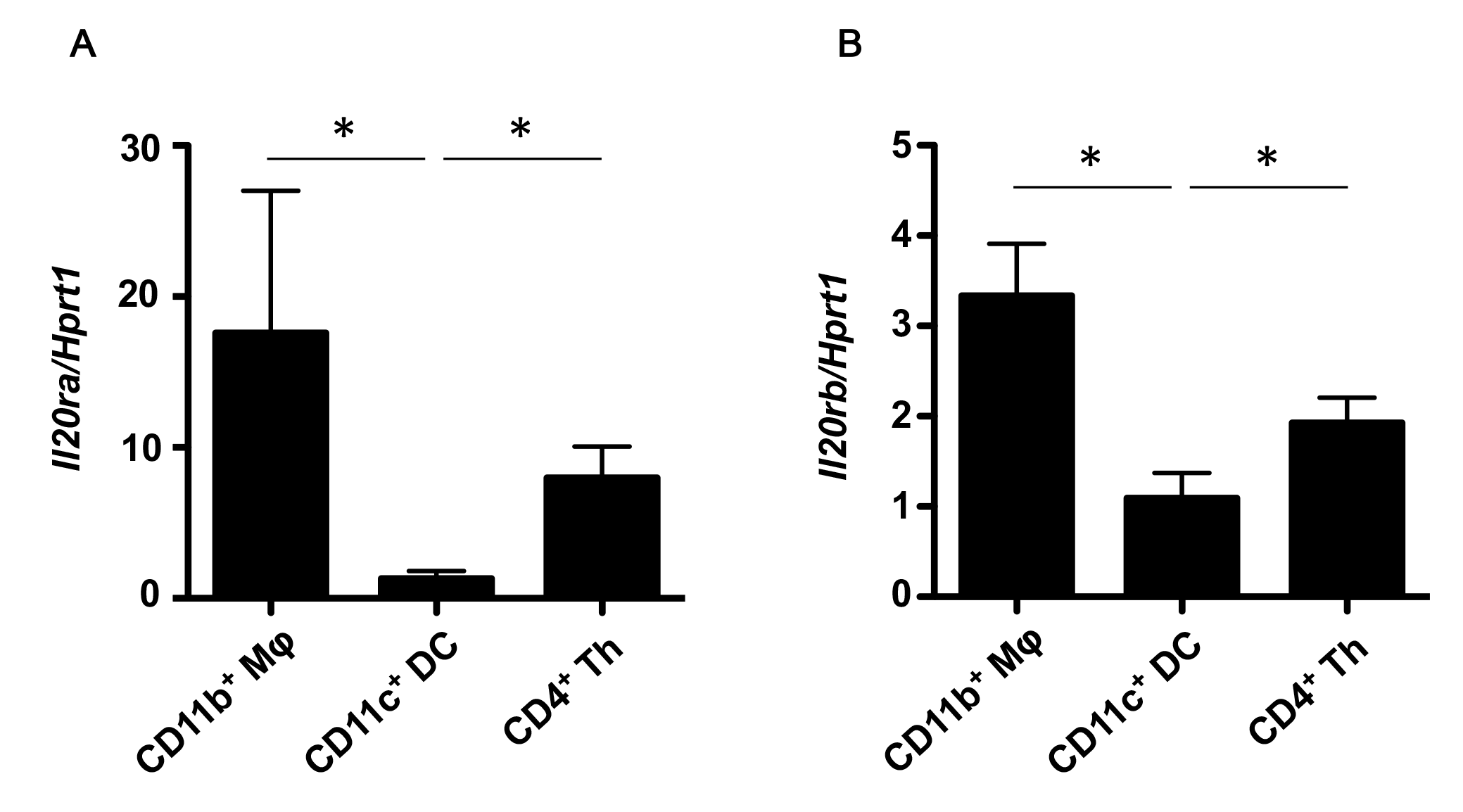

Supplement: Supplementary file 2 [file Image_2.TIF]

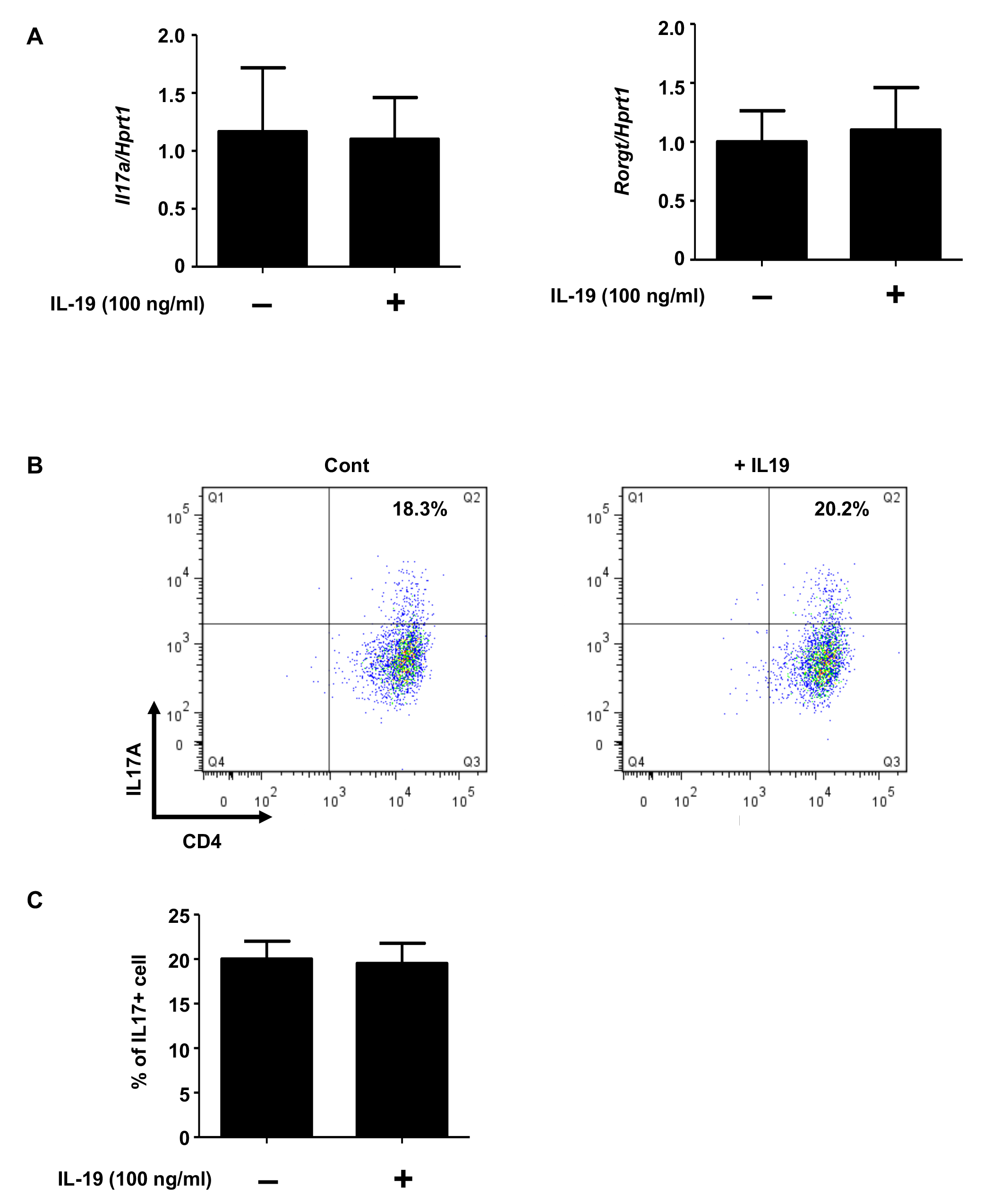

Supplement: Supplementary file 3 [file Image_3.TIF]

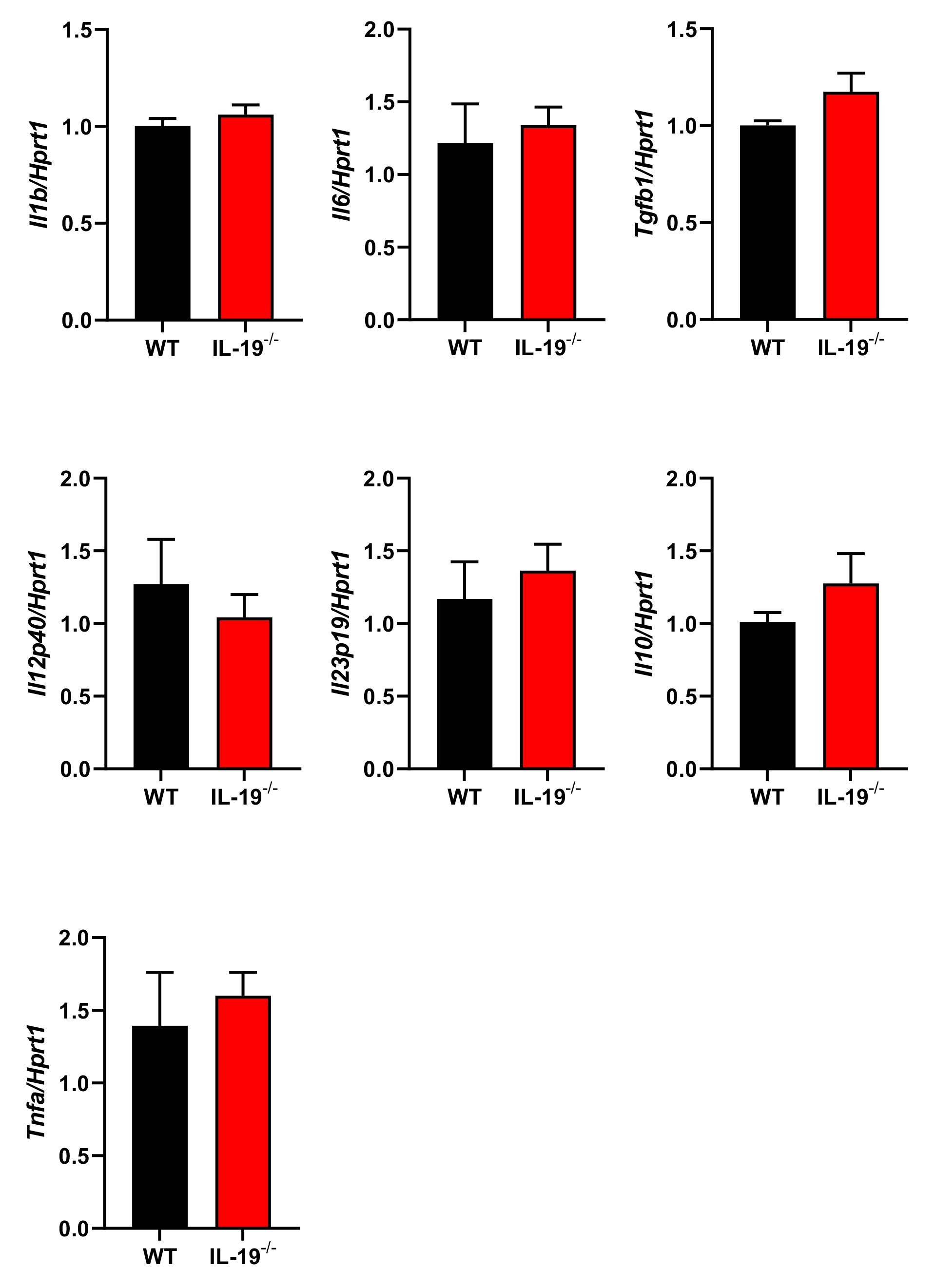

Supplement: Supplementary file 4 [file Image_4.TIF]
