# Supplementary material for: Interleukin-19 Abrogates Experimental Autoimmune Encephalomyelitis by Attenuating Antigen-Presenting Cell Activation
Source: Front Immunol. 2021 Mar 11;12:615898. doi: 10.3389/fimmu.2021.615898 (PMC7990911; doi:10.3389/fimmu.2021.615898)
Supplement: Supplementary file 5 [file Data_Sheet_1.docx]

**Supplementary Figure Legends**

**Figure S1. IL-19 deficient mice without immunization did not show inflammatory cell infiltration in the CNS.**

Micrographs of hematoxylin/eosin staining of L5 lumbar spinal cords of WT and IL-19^-/-^ mice without immunization. The bottom panels show enlargements of the boxed areas in the upper panels. Scale bars: 500 μm (upper), 100 μm (bottom). (B) Quantitative analysis of cell infiltration in L5 lumbar spinal cords without EAE (n = 4). ND, not detected.

**Figure S2. IL-19 receptor heterodimer subunits IL-20Rα and IL-20Rβ are more highly expressed in macrophage and helper T cells than in dendritic cells.**

(A) qPCR for mRNA encoding IL-20Rα in CD11b^+^ macrophages, CD11c^+^ dendritic cells, and CD4^+^ helper T cells in the spleen. (B) qPCR data for mRNA encoding IL-20Rβ in CD11b^+^ macrophages, CD11c^+^ dendritic cells, and CD4^+^ T cells in the spleen. Data are represented as means ± SEM. *, *p* < 0.05 (n = 3).

**Figure S3. IL-19 does not alter differentiation of naïve T cells into Th17 cells.**

(A) qPCR data for mRNAs encoding IL-17A and RORγt. (B) Representative flow cytometric data for IL-17A expression. (C) Quantitative analysis of (B). Data are represented as means ± SEM. *, *p* < 0.05 (n = 5).

**Figure S4. IL-19 deficiency does not alter the expression levels of Th17 cell differentiation–associated cytokines in dendritic cells.**

qPCR data for mRNAs encoding IL-1β, IL-6, TGF-β1, IL-12 p40, IL-23 p19, IL-10, and TNF-α expression in splenic dendritic cells of EAE mice. Assessments were performed 7 days after immunization. Data are represented as means ± SEM (n = 6).
